# From Basic Research to Molecular Breeding — Chinese Scientists Play A Central Role in Boosting World Rice Production

**DOI:** 10.1016/j.gpb.2018.12.002

**Published:** 2018-12-27

**Authors:** Ding Tang, Zhukuan Cheng

**Affiliations:** State Key Laboratory of Plant Genomics and Center for Plant Gene Research, Institute of Genetics and Developmental Biology, Chinese Academy of Sciences, Beijing 100101, China

## Abstract

On November 18, 2018, the Future Science Prize Awarding Ceremony was held in Beijing. In the area of life science, Professors Jiayang Li, Longping Yuan, and Qifa Zhang shared the prize for their pioneering contributions in producing high-yield, superior-quality rice through systematic study of molecular mechanisms associated with specific rice features and application of novel approaches in rice breeding. The Future Science Prize is also touted as “China’s Nobel Prize”, fully affirming their achievements in rice basic research and breeding.

The 2018 China’s Future Science Prize in Life Science was jointly awarded to Profs. Jiayang Li, Longping Yuan, and Qifa Zhang, in recognition of their groundbreaking discoveries leading to the development of innovative tools for breeding high-yield and superior-quality rice varieties (Figure 1). The Future Science Prize is one of China’s highly regarded awards established in 2016 (http://futureprize.org/), being touted as the Chinese version of the Nobel Prize.

**Figure 1:**
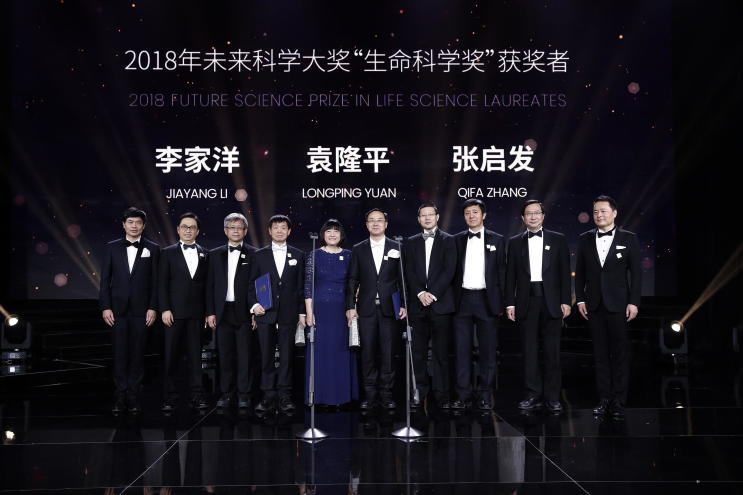
**A photo of winners, Science Committee Members, and Donors of the 2018 Future Science Prize in Life Science during the Awarding Ceremony on November 18, 2018, in Beijing**

Rice is the staple food for more than half of the world’s population, and in China, over 60% of its 1.4 billion people consume rice on a daily basis. The dramatic increase in population coupled with global climate change, reduced agricultural land, and environmental pollution pose a big challenge for food security in China. As such, increasing rice production is critical to sustain and improve people’s livelihood, national economy, and even national security. Over the past six decades, China has made extraordinary accomplishments in boosting its rice production. Rice yield has experienced at least two quantum jumps; the first was brought by dwarf breeding, the so-called “Green Evolution” in the 1960s, and the second came from the introduction of hybrid rice in the 1970s.

The Green Revolution has dramatically increased crop production, thanks to the development of high-yield varieties through deployment of semi-dwarf genes in rice and wheat. The rice semi-dwarf gene *sd1* was first identified from the Chinese rice cultivar named “Dee-geo-woo-gen” and since then has been widely bred into the current rice varieties [1]. Guang-chang-ai is the first of its kind that was developed in China through the introduction of the *sd1* gene [2]. The semi-dwarf varieties accounted for 20%–30% yield increase when compared with the conventional ones because of their high harvest index, resistance to lodging, and improved response to fertilizers [3]. Developing rice dwarf varieties has been widely considered as one of the most important achievements in rice breeding history.

Another breakthrough achievement was to harness heterosis by developing and growing hybrid rice. Heterosis or hybrid vigor refers to a situation in which the hybrids perform better than their parents, and this has been exploited to improve crop production for nearly a century [4]. Exploitation and utilization of heterosis in rice was first initiated by Prof. Longping Yuan in the 1960s, and a significant progress was made in 1970 due to the discovery of a cytoplasmic male sterility (CMS) line from wild rice (*Oryza rufipogon*) [5]. Five years later, large-scale hybrid seed production using a three-line system was fully established, making it feasible to commercially produce hybrid rice [6]. Several additional CMS lines were later identified and successively exploited [7], which had greatly expanded the germplasm pool of CMS. The subsequent establishment of the two-line hybrid system broadened the use of hybrid vigor both within and between subspecies, and this technology further increased rice yield by 5%–10% compared to the three-line system [6]. In 1996, the Chinese government launched a nationwide “Super Rice Breeding Program”, with an ultimate goal to further boost rice yield through an improved understanding of the theory and practice of hybrid development. In recent field tests, Super Hybrid Rice has set a new world record by reaching an average yield over 1000 kg per mu (about 0.07 ha). The Super Hybrid Rice is characterized by its ideal plant architecture (ideotype) and utilization of the inter-subspecific heterosis [8]. After more than 40 years of application, hybrid rice has become one of the greatest innovations in agriculture, making a massive contribution to food security in both China and the world.

China has been a major player of the rice genome research, contributing to sequencing and resequencing genomes of many cultivated and wild rice varieties [9]. A wealth of genomic data combined with fast-growing biotechnologies greatly facilitated gene discovery and functional analyses. Prof. Jiayang Li and his team successfully cloned *MONOCULM 1* (*MOC1*), a key regulator controlling rice tiller number [9]. Thereafter, Chinese scientists have made great strides in isolating dozens of key genes relevant to important agronomic traits. Examples of such genes include the plant architecture controlling genes (*IPA1* [10], *PROG1* [11], and *D53* [12]), panicle architecture related genes (*DEP1* [13] and *NOG1* [14]), grain size controlling genes (*GS2* [15], *GS3* [16], *GS5* [17], and *GW5* [18]), rice grain quality genes (*Wx* [19], *ALK* [20], and *Badh2* [21]), cold resistance genes (*COLD1* [22], *CTB4a* [23], and *LTG1* [24]), heat tolerance genes (*TT1* [25] and *HTAS* [26]), salt tolerance gene (*SKC1* [27]), drought resistance gene (*DWA1* [28]), disease resistance genes (*STV11* [29], *PIGM* [30], *Bsr-d1* [31], and *Xa4* [32]), insect resistance genes (*Bph3* [33] and *Bph14* [34]), heading date genes (*GHD7* [35] and *GHD8* [36]), nitrogen nutrient efficiency gene (*NRT1.1B* [37]), and photoperiodic sensitive male sterile gene (*PMS3* [38]). Moreover, Chinese scientists have also made significant breakthroughs in elucidating the genetic and molecular mechanisms underlying rice heterosis [39], [40], CMS [41], and fertility between *indica* and *japonica* varieties [42], [43].

With so many genes mapped or cloned, it is pivotal to design a molecular strategy to breed better rice varieties that require less input and can adapt to various environmental constraints. This is particularly helpful for the smallholder farmers in sub-Saharan Africa and Asia who grow crops under stress conditions but have limited financial resources. For this purpose, Prof. Qifa Zhang, together with researchers from the International Rice Research Institute (IRRI) and Chinese Academy of Agricultural Sciences (CAAS) funded by the Bill and Melinda Gates Foundation, put forward a long-term strategy to develop the so-called Green Super Rice (GSR). GSR is a new strategy for generating high-yield varieties and hybrids that are tolerant to various abiotic stresses such as drought, floods, and salinity, resistant to multiple pests and diseases, and with high nitrogen and phosphorus use efficiency and superior nutritional quality [44]. After ten years of continuous efforts, the GSR program has achieved encouraging progresses. As of March 2018, 75 new GSR varieties have been developed, with total planting area exceeding 6.67 million hectare [45].

Breeding by design aims to bring together favorable alleles of all agronomically important genes into a single genotype [46]. This concept has driven the development of frontier technologies of crop breeding in China [47], [48], [49]. As an active advocator and practitioner, Prof. Jiayang Li and his colleagues have developed a series of well-designed lines through the “breeding by molecular design” approach. Examples of the germplasm include Jiayouzhongke series, Zhongkefa series, and Zhongke804, which possess high yield, superior quality, disease and lodging resistance, and resilience to environmental stresses [50]. Prof. Li’s seminal work lays a solid foundation for future rice improvement.

## References

[1] Hedden P. (2003). The genes of the Green Revolution. Trends Genet.

[2] Gu M. (2010). Discussion on the aspects of high-yielding breeding in rice. Acta Agron Sin.

[3] Yuan L. (1996). Prospects for yield potential in rice through plant breeding. Hybrid Rice.

[4] Fujimoto R., Uezono K., Ishikura S., Osabe K., Peacock W.J., Dennis E.S. (2018). Recent research on the mechanism of heterosis is important for crop and vegetable breeding systems. Breed Sci.

[5] Yuan L. (1986). Chinese hybrid rice. China Rice Sci.

[6] Ma G., Yuan L. (2015). Hybrid rice achievements, development and prospect in China. J Integr Agric.

[7] Ma G., Yuan L. (2003). Hybrid rice achievements and development in China. Hybrid rice for food security, poverty alleviation, and environmental protection.

[8] Yuan L., Denning G.L., Mew T.W. (1998). Hybrid rice breeding for super high yield. China and IRRI: improving China's rice productivity in the 21st century.

[9] Li X., Qian Q., Fu Z., Wang Y., Xiong G., Zeng D. (2003). Control of tillering in rice. Nature.

[10] Jiao Y., Wang Y., Xue D., Wang J., Yan M., Liu G. (2010). Regulation of OsSPL14 by OsmiR156 defines ideal plant architecture in rice. Nat Genet.

[11] Tan L., Li X., Liu F., Sun X., Li C., Zhu Z. (2008). Control of a key transition from prostrate to erect growth in rice domestication. Nat Genet.

[12] Jiang L., Liu X., Xiong G., Liu H., Chen F., Wang L. (2013). DWARF 53 acts as a repressor of strigolactone signalling in rice. Nature.

[13] Huang X. (2009). Natural variation at the DEP1 locus enhances grain yield in rice. Nat Genet.

[14] Huo X., Wu S., Zhu Z., Liu F., Fu Y., Cai H. (2017). NOG1 increases grain production in rice. Nat Commun.

[15] Hu J., Wang Y., Fang Y., Zeng L., Xu J., Yu H. (2015). A rare allele of GS2 enhances grain size and grain yield in rice. Mol Plant.

[16] Mao H., Sun S., Yao J., Wang C., Yu S., Xu C. (2010). Linking differential domain functions of the GS3 protein to natural variation of grain size in rice. Proc Natl Acad Sci U S A.

[17] Li Y., Fan C., Xing Y., Jiang Y., Luo L., Sun L. (2011). Natural variation in GS5 plays an important role in regulating grain size and yield in rice. Nat Genet.

[18] Weng J., Gu S., Wan X., Gao H., Guo T., Su N. (2008). Isolation and initial characterization of GW5, a major QTL associated with rice grain width and weight. Cell Res.

[19] Wang Z.Y., Wu Z.L., Xing Y.Y., Zheng F.G., Guo X.L., Zhang W.G. (1990). Nucleotide sequence of rice waxy gene. Nucleic Acids Res.

[20] Gao Z., Zeng D., Cui X., Zhou Y., Yan M., Huang D. (2003). Map-based cloning of the ALK gene, which controls the gelatinization temperature of rice. Sci China C Life Sci.

[21] Chen S., Yang Y., Shi W., Ji Q., He F., Zhang Z. (2008). *Badh2*, encoding betaine aldehyde dehydrogenase, inhibits the biosynthesis of 2-acetyl-1-pyrroline, a major component in rice fragrance. Plant Cell.

[22] Ma Y., Dai X., Xu Y., Luo W., Zheng X., Zeng D. (2015). *COLD1* confers chilling tolerance in rice. Cell.

[23] Zhang Z., Li J., Pan Y., Li J., Zhou L., Shi H. (2017). Natural variation in *CTB4a* enhances rice adaptation to cold habitats. Nat Commun.

[24] Lu G., Wu F.Q., Wu W., Wang H.J., Zheng X.M., Zhang Y. (2014). Rice LTG1 is involved in adaptive growth and fitness under low ambient temperature. Plant J.

[25] Li X.M., Chao D.Y., Wu Y., Huang X., Chen K., Cui L.G. (2015). Natural alleles of a proteasome α2 subunit gene contribute to thermotolerance and adaptation of African rice. Nat Genet.

[26] Liu J., Zhang C., Wei C., Liu X., Wang M., Yu F. (2016). The RING finger ubiquitin E3 ligase OsHTAS enhances heat tolerance by promoting H_2_O_2_-induced stomatal closure in rice. Plant Physiol.

[27] Ren Z.H., Gao J.P., Li L.G., Cai X.L., Huang W., Chao D.Y. (2005). A rice quantitative trait locus for salt tolerance encodes a sodium transporter. Nat Genet.

[28] Zhu X., Xiong L. (2013). Putative megaenzyme DWA1 plays essential roles in drought resistance by regulating stress-induced wax deposition in rice. Proc Natl Acad Sci U S A.

[29] Wang Q., Liu Y., He J., Zheng X., Hu J., Liu Y. (2014). *STV11* encodes a sulphotransferase and confers durable resistance to rice stripe virus. Nat Commun.

[30] Deng Y., Zhai K., Xie Z., Yang D., Zhu X., Liu J. (2017). Epigenetic regulation of antagonistic receptors confers rice blast resistance with yield balance. Science.

[31] Li W., Zhu Z., Chern M., Yin J., Yang C., Ran L. (2017). A natural allele of a transcription factor in rice confers broad-spectrum blast resistance. Cell.

[32] Hu K., Cao J., Zhang J., Xia F., Ke Y., Zhang H. (2017). Improvement of multiple agronomic traits by a disease resistance gene via cell wall reinforcement. Nat Plants.

[33] Liu Y., Wu H., Chen H., Liu Y., He J., Kang H. (2015). A gene cluster encoding lectin receptor kinases confers broad-spectrum and durable insect resistance in rice. Nat Biotechnol.

[34] Du B., Zhang W., Liu B., Hu J., Wei Z., Shi Z. (2009). Identification and characterization of *Bph14*, a gene conferring resistance to brown planthopper in rice. Proc Natl Acad Sci U S A.

[35] Xue W., Xing Y., Weng X., Zhao Y., Tang W., Wang L. (2008). Natural variation in *Ghd7* is an important regulator of heading date and yield potential in rice. Nat Genet.

[36] Yan W.H., Wang P., Chen H.X., Zhou H.J., Li Q.P., Wang C.R. (2011). A major QTL, *Ghd8*, plays pleiotropic roles in regulating grain productivity, plant height, and heading date in rice. Mol Plant.

[37] Hu B., Wang W., Ou S., Tang J., Li H., Che R. (2015). Variation in *NRT1.1B* contributes to nitrate-use divergence between rice subspecies. Nat Genet.

[38] Ding J., Lu Q., Ouyang Y., Mao H., Zhang P., Yao J. (2012). A long noncoding RNA regulates photoperiod-sensitive male sterility, an essential component of hybrid rice. Proc Natl Acad Sci U S A.

[39] Huang X., Yang S., Gong J., Zhao Q., Feng Q., Zhan Q. (2016). Genomic architecture of heterosis for yield traits in rice. Nature.

[40] Huang X., Yang S., Gong J., Zhao Y., Feng Q., Gong H. (2015). Genomic analysis of hybrid rice varieties reveals numerous superior alleles that contribute to heterosis. Nat Commun.

[41] Luo D., Xu H., Liu Z., Guo J., Li H., Chen L. (2013). A detrimental mitochondrial-nuclear interaction causes cytoplasmic male sterility in rice. Nat Genet.

[42] Yang J., Zhao X., Cheng K., Du H., Ouyang Y., Chen J. (2012). A killer-protector system regulates both hybrid sterility and segregation distortion in rice. Science.

[43] Yu X., Zhao Z., Zheng X., Zhou J., Kong W., Wang P. (2018). A selfish genetic element confers non-mendelian inheritance in rice. Science.

[44] Zhang Q. (2007). Strategies for developing green super rice. Proc Natl Acad Sci U S A.

[45] Zhang C.P., Yu S.B., Zhang Q.F. (2018). Recent advances in green super rice development. Chin Bull Life Sci.

[46] Peleman J.D., van der Voort J.R. (2003). Breeding by design. Trends Plant Sci.

[47] Qian Q., Guo L., Smith S.M., Li J. (2016). Breeding high-yield superior quality hybrid super rice by rational design. Natl Sci Rev.

[48] Wang Y., Xue Y., Li J. (2005). Towards molecular breeding and improvement of rice in China. Trends Plant Sci.

[49] Zeng D., Tian Z., Rao Y., Dong G., Yang Y., Huang L. (2017). Rational design of high-yield and superior-quality rice. Nat Plants.

[50] Yu H., Wang B., Chen M., Liu G., Li J. (2018). Research advance and perspective of rice breeding by molecular design. Chin Bull Life Sci.

